# Bacteria detected in the genital tract, semen or pre-ejaculatory fluid of Swedish stallions from 2007 to 2017

**DOI:** 10.1186/s13028-019-0459-z

**Published:** 2019-05-30

**Authors:** Ziyad Al-Kass, Erik Eriksson, Elisabeth Bagge, Margareta Wallgren, Jane Margaret Morrell

**Affiliations:** 1 Department of Clinical Sciences, Swedish University of Agricultural Sciences, Box 7054, 75007 Uppsala, Sweden; 2 Department of Bacteriology, National Veterinary Institute, 751 89 Uppsala, Sweden; 3 Department of Surgery and Theriogenology, College of Veterinary Medicine, University of Mosul, Mosul, Iraq

**Keywords:** Antibiotics, Equine insemination, Microbial flora, Potential pathogens, Semen quality

## Abstract

**Background:**

Although artificial insemination (AI) was developed as a means of controlling disease transmission, pathogens can still be transmitted to females in semen used for AI. In addition, bacteria can cause deterioration in sperm quality during storage. Semen becomes contaminated by the male’s normal bacterial flora as it passes out of the reproductive tract but potential pathogens may also contaminate the semen. Therefore, semen samples from stallions to be used for AI are tested before the breeding season to minimize transmission of pathogens to inseminated mares. In Sweden, semen samples are tested at the National Veterinary Institute, Uppsala (SVA). For the present study, a retrospective analysis was made of potentially pathogenic bacteria isolated from samples submitted to the SVA from 2007 to 2017.

**Results:**

In our study, *Taylorella equigenitalis* was found infrequently (53 out of 25,512 samples), representing 11 out of 2308 stallions. If *T. equigenitalis* was detected, the stallions were treated with antibiotics and re-tested later in the same year. *Klebsiella pneumoniae* and beta haemolytic streptococci were the most commonly found potential pathogens, whereas *Pseudomonas aeruginosa* was also isolated occasionally. There were considerable differences in the number of species isolated each year.

**Conclusions:**

Potential pathogens were identified in relatively few of the samples submitted to SVA during this period, with *T. equigenitalis* not being identified since 2015. Of the other potential pathogens, *K. pneumoniae* and beta haemolytic streptococci were the most common. The information is relevant for determining guidelines on the testing and treatment of stallions before breeding.

## Background

Although artificial insemination (AI) was first developed as a means of controlling disease transmission, pathogens can still be transmitted in semen used for AI. These pathogens may infect inseminated females or contribute to a rapid deterioration in sperm quality.

Semen of healthy stallions is considered to be sterile when produced, but it becomes contaminated by microorganisms as it passes through the genital tract at ejaculation. Furthermore, semen collected for AI may become contaminated from the air, from equipment used in semen collection and during handling [1], from the semen collector and by microorganisms naturally present on the mucosal membranes or skin of the penis and prepuce [2].

In addition to the normal flora, pathogens such as *Pseudomonas aeruginosa* [3], *Klebsiella pneumoniae* [4, 5] and *Streptococcus equi* subsp*. zooepidemicus* [6] are occasionally found. *Taylorella equigenitalis* may also be present [7]. Due to the importance of *T. equigenitalis* infection in breeding horses, it is compulsory for stallions at AI centers in Sweden to be tested before the start of the breeding season. The presence of *T. equigenitalis* is notifiable.

Antibiotics are commonly added to semen extenders to reduce the growth of bacteria that may have contaminated the ejaculate during semen collection. However, this practice represents a non-therapeutic use of antibiotics, which may contribute to the development of antibiotic resistance. Since the reproductive tract of the mare has a well-developed immune defense to eliminate bacteria deposited during natural mating, small numbers of bacteria in inseminated semen may not represent a source of infection. Therefore, increasing awareness of the type of bacteria likely to occur in semen may enable a more prudent use of antibiotics to be suggested, in line with the current need to reduce antibiotic usage.

The aim of this study was to analyse bacteriological findings of genital tract swabs, semen and pre-ejaculatory fluids submitted to the National Veterinary Institute in Uppsala, Sweden (SVA) over a decade, to determine the prevalence of bacterial genital tract pathogens present in Swedish stallions.

## Methods

### Samples

During the period 2007 to 2017, 25,512 samples originating from 2308 stallions of various breeds and ages in Sweden were taken by veterinarians and submitted to SVA for testing specifically for *T. equigenitalis*. The samples consisted of swabs of the penile shaft or prepuce, urethral fossa, urethral orifice and semen or pre-ejaculatory fluid, although not all sites were sampled from all animals. Veterinarian were instructed to pass a cotton swab over the skin folds of the prepuce, urethra, urethral fossa, semen and/or the pre-ejaculatory fluid. The swabs were submitted to the laboratory in Amies charcoal transport medium within 48 h after sampling. Of the 2308 stallions, 2099 were sampled on more than once occasion, since animals from which *T. equigenitalis* was detected in any of the samples were treated and re-tested; in some instances, samples were taken on multiple occasions in the same year.

In addition, results were available from 730 semen samples or pre-ejaculatory fluid from 319 stallions in Sweden submitted to SVA for general bacteriological analysis during the same period. Data were compiled from routine analysis for detection of presumptive pathogenic bacteria such as *P. aeruginosa*, *K. pneumoniae* and beta haemolytic streptococci. These semen samples were separate from those used for testing for *T. equigenitalis*.

### Bacteriological culture and identification

#### Examination for *Taylorella equigenitalis*

Upon arrival at the laboratory swabs were plated on three different haematin “chocolate” blood agar, one with no inhibitors, one containing streptomycin sulphate (200 µg/mL) and the third one with 1 µg/mL trimethoprim, 5 µg/mL clindamycin and 5 µg/mL amphotericin B. All culture media were prepared by SVA. The plates were incubated in CO_2_ at 37 °C for 7 days. Any 0.8–1.0 mm wide white and peaked colonies on blood agar containing Gram-negative rods, and producing catalase and oxidase, were suspected of being *T. equigenitalis*. Further identification were performed by API zym kits (BioMerieux, USA) [8], Mono-Tayl-agglutinations test (BioNor, Norway) and ALA-test. From 2012, *T. equigenitalis* was identified by matrix assisted laser desorption ionization time-of-flight mass spectrometry (MALDI-TOF MS).

#### General bacteriological analysis

Samples of semen and pre-ejaculatory fluid were cultured on horse blood agar, bromocresole lactose purpur agar and *Pseudomonas* isolation agar (SVA, produced in-house) and incubated at 37 °C for 48 h. The agar plates were examined after both 24 and 48 h’ incubation. Presumptive pathogenic bacterial colonies were re-cultured on horse blood agar to obtain monocultures for further identification. Only the presence of potentially pathogenic bacteria, such as *Klebsiella* spp. and *Pseudomonas* spp., were recorded in the data set; however, the presence of *Enterobacter* spp. was also reported because of its resemblance to *Klebsiella* spp. In the case that potential pathogens were detected, the organism was identified, together with a susceptibility test for the isolated pathogen. If no pathogenic bacteria were identified, the type of growth was recorded as either (i) no bacterial growth, (ii) growth of unspecific mixed flora, or (iii) sporadic, sparse, moderate or rich growth of unspecified mixed flora.

The methods used for bacterial identification at SVA have changed over the years. Before 2012, bacteriological identification was based on traditional methods, relying on morphological/macroscopic features on different agars, phenotypic identification using Gram-staining, culturing and simple biochemical tests for instance, catalase, oxidase, indole, urease, coagulase and API 20E (BioMérieux, Marcy-l’Étoile, France). After 2012 all identification to species level was performed using MALDI-TOF MS (Bruker Daltonics, Bremen, Germany) [9].

## Results

### Examination for *Taylorella equigenitalis*

The samples originated from 71 studs around the country, mostly from the middle and south of Sweden. The distribution of material provided for testing is shown in Table 1.

**Table 1:** Distribution of samples provided for testing for *Taylorella equigenitalis*, 2007–2017

Swabs, semen or pre-ejaculatory fluid	Number
Penile shaft/prepuce + urethral fossa + urethral orifice + semen	3555
Penile shaft/prepuce + urethral fossa + urethral orifice	3520
Semen	231
Other combinations or single samples	501
Total (different combinations or single samples)	7807
Total (individual samples)	25,512

Of the 2308 stallions tested, *T. equigenitalis* was detected from 53 swabs taken from 11 stallions (Table 2); 14 additional swabs taken at the same time from the same stallions were negative (Table 3). Infected animals were re-tested after treatment; therefore, some animals were tested repeatedly during any 1 year. At the post-treatment sampling in the same year, *T. equigenitalis* was isolated again from four stallions but was not isolated on subsequent samplings later in the year or in subsequent years. Two additional stallions (stallions 5 and 6, Table 3) appeared to have responded to treatment, since *T. equigenitalis* was not isolated from a set of swabs taken after treatment, but the organism was found again at subsequent testing later in the same year. For one of these two stallions (stallion 6), *T. equigenitalis* was not found subsequently after the 3rd and 4th testing, despite repeated samplings (10 sets of swabs) over 2 years. However, for stallion 5, *T. equigenitalis* was isolated from the first set of swabs, was not isolated from the 2nd, 3rd and 4th sets of swabs but was isolated from the 5th and 6th set of swabs. It is not known what became of this stallion afterwards as no further swabs were submitted to SVA for testing. For two of the stallions (9 and 11 in Table 3) *T. equigenitalis* was isolated in 2011 and 2010 respectively, despite not having been isolated in previous years.

**Table 2:** Distribution of samples and results of the analysis of detection of *Taylorella equigenitalis* in stallions in Sweden according to year

Year	Stallions included/year	*T. equigenitalis* isolated, no. of stallions	Total no. of samples	*T. equigenitalis* isolated, no. of samples
2007	511	3 (0.6%)	2434	20 (0.8%)
2008	494	1 (0.2%)	2504	2 (0.1%)
2009	485	3 (0.6%)	2518	15 (0.6%)
2010	532	2 (0.4%)	2570	5 (1.2%)
2011	478	1 (0.2%)	2410	7 (0.3%)
2012	482	0 (0.0%)	2556	0 (0.0%)
2013	452	0 (0.0%)	2381	0 (0.0%)
2014	480	0 (0.0%)	2471	0 (0.0%)
2015	469	1 (0.2%)	2111	4 (0.2%)
2016	424	0 (0.0%)	1804	0 (0.0%)
2017	423	0 (0.0%)	1753	0 (0.0%)
Total	5230	11 (0.2%)	25,512	53 (0.2%)

**Table 3:** Pattern of isolation of *Taylorella equigenitalis* from various sampling sites

Stallion	Year	Occasion	Penis shaft/prepuce	Urethral fossa	Urethral orifice	Semen/pre-ejaculate
1	2007	1st testing	+	+	+	+
		2nd testing	+	+	+	+
	2007–2011	Multiple testings (n = 4)	−	−	−	−
2	2007	1st testing	+	+	+	+
	2007–2015	Multiple testings (n = 36)	−	−	−	−
3	2007	1st testing	+	+	+	+
		2nd testing	+	+	+	+
	2007–2016	Multiple testings (n = 14)	−	−	−	−
4	2008	1st testing	+	+	−	−
	2008–2017	Multiple testings (n = 15)	−	−	−	
5	2009	1st testing	+	+	+	
	2009	2nd–4th testing	−	−	−	
	2009	5th + 6th testing	−	+	+	
6	2009	1st + 3rd testing		+		
	2009	2nd testing	−	−	−	
	2009–2011	Multiple testings (n = 10)	−	−	−	
7	2009	1st testing	−	+	+	+
	2009–2015	Multiple testings (n = 9)	−	−	−	−
8	2010	1st testing	+	−	+	
	2010–2011	Multiple testings (n = 2)	−	−	−	
9	2007	Multiple testings (n = 2)	−	−	−	−
	2011	1st testing	+	+	+	+
	2011	2nd testing	−	+	+	+
	2011–2017	Multiple testings (n = 17)	−	−	−	−
10	2015	1st testing	+	+	+	
		2nd testing	−	+	−	
		3rd testing	−	−	−	
11	2007–2008	Multiple testings (n = 4)	−	−	−	
	2010	1st testing	−	+	+	+
	2010–2015	Multiple testings (n = 11)	−	−	−	−

+: *Taylorella equigenitalis* isolated; −: *Taylorella equigenitalis* not isolated; blank square: no sample submitted

*Taylorella equigenitalis* was identified from 11 out of 19 swabs from the penile shaft and prepuce, 18 out of 19 swabs from the urethral fossa, 15 out of 19 swabs from the urethral orifice, and 9 out of 10 samples from semen/pre-ejaculatory fluid. For six of the stallions, *T. equigenitalis* was found on two or more sampling occasions (Table 3). Nine of the positive animals that became negative after treatment were tested in subsequent years (between 2 and 10 years, depending on the stallion). The results remained negative for these animals.

### General bacteriological analysis

The 730 semen samples submitted for general bacteriological analyses originated from 319 stallions. The number of samples analysed per year (Table 4) was highest in 2008 (n = 145) and lowest in 2017 (n = 9). Three potentially pathogenic species were detected (Table 5), with *K. pneumoniae*, beta haemolytic streptococci and *Pseudomonas* spp. being isolated from 8, 8 and 3 animals, respectively.

**Table 4:** Distribution of mixed bacterial flora in samples and animals according to year

Year	No. of semen samples	Animals per year	No. of samples	No. of animals
2007	65	57	1	1
2008	145	72	7	3
2009	141	87	3	3
2010	127	85	2	2
2011	40	40	3	3
2012	70	66	3	2
2013	41	38	1	1
2014	36	35	1	1
2015	35	27	6	4
2016	21	20	4	4
2017	9	8	3	3
Total	730	319^a^	34	27

In some cases, several samples originated from the same animal

^a^Number of individuals

**Table 5:** Potential pathogens detected in stallion genital tract samples 2007–2017

Bacterial species	No. of samples	No. of animals
*Klebsiella pneumoniae*	15	8
Beta haemolytic streptococci	10	8
*Pseudomonas aeruginosa*	7	3
Total	32	19

In most of the cultures, growth of mixed flora was observed and the bacteria were considered to be non-pathogenic. Therefore in these cases, a report of “No specific infection” was made, together with an estimate of the amount of growth of mixed flora. The classification and distribution of bacteria in the samples where no specific infection was identified are shown in Table 6.

**Table 6:** Semiquantitative scoring of bacterial growth in cases where mixed flora (no specific infection) was

Semiquantitative scoring	No. of samples
Sparse growth of mixed flora	322
Moderate growth of mixed flora	174
Rich growth of mixed flora	44
Sporadic growth^a^ of mixed flora	13
A single colony	5
No growth of bacteria	125

^a^2–5 colonies of different species

## Discussion

In this study, a retrospective data analysis was made of potentially pathogenic bacteria isolated from samples of the stallion reproductive tract submitted to SVA during the period 2007 to 2017. Most of the samples originated from stallions in the middle and southern parts of Sweden, reflecting the distribution of the human population.

There was a lower prevalence of *T. equigenitalis* in stallions in Sweden than in some other countries. In a study from the USA, *T. equigenitalis* was detected in 22 out of 222 stallions in the period 2008 to 2010 [10]. In contrast, this organism was detected in four out of 120 imported stallions entering Darlington, Maryland, USA, between 1999 and 2001 [11]. Furthermore, it was found in 17 out of 245 stallions in Slovenia, (6.9%) [12] while the rate of detection in the Republic of Korea has decreased from 13.5% in 2015 to 1.0% in 2017 [13].

*Pseudomonas aeruginosa*, *K. pneumoniae* and beta haemolytic streptococci were found in 5% of samples submitted for general bacteriological examination. *K. pneumoniae* were isolated from 2.5% (11/319) of the sampled animals, which is fewer than in a previous Swedish study [2] where *Klebsiella* spp. were isolated from 4% (5/115) of the examined horses. In other studies, *Klebsiella* spp. have been isolated more frequently, although in much smaller sample sizes. In a Spanish study, Ortega-Ferrusola et al. [﻿14] detected *Klebsiella* spp. in 3 out of 5 semen samples, whereas Spergser et al. [15﻿] detected *Klebsiella* spp. from 13 out of 39 stallions in a study from the northwestern region of Austria.

Beta haemolytic streptococci were isolated infrequently (10/319 animals), in concordance with other studies. Previous reports showed these bacteria in only 7 out 116 of sampled stallions [15], 1 out of 20 stallions [16] and 14 of 115 samples [2], respectively. In contrast, there are also studies that report beta haemolytic streptococci to be the most frequently isolated bacteria, for instance in 4 out of 5 stallions [14].

In a Swedish study performed 20 years ago [2], 18 species of bacteria were found, of which five were also detected in our study. The most frequently isolated species in the previous study were *Pseudomonas* spp. (56/115), *Enterobacter* spp. (41/115) and *Bacillus* spp. (31/115) [2]. In comparison, in the present study, *P. aeruginosa* was detected in 4 of 319 animals. No screening for *Bacillus* spp. or *Enterobacter* spp. was done as they are not considered to be potentially pathogenic in horses.

Although these results could indicate a change in the microflora, there could also be other explanations, such as that methods for culture and identification have changed in the intervening period since the previous study in Sweden. However, it was reassuring to note that few potential pathogens were identified in the present study. Methodological differences and differences in sampling sites probably also account for the variations between our results and similar studies in other countries [2, 17, 18].

## Conclusion

Identification of *T. equigenitalis* and treatment of the carrier stallions appears to be effective in preventing transmission of the organism, since the organism was found in only one stallion since 2012. Other potential pathogens were identified in relatively few of the samples submitted to SVA during this period, with *K. pneumoniae* and beta haemolytic streptococci being the most commonly isolated. There were differences in the prevalence of specific bacteria in various studies in other countries differences in the occurrence of various bacteria, although this may be due, in part, to differences in the methodologies used for identification of bacteria.

## Data Availability

The datasets used and/or analysed during the current study are available from the corresponding author on reasonable request, in relation to the Swedish data protection act.
